# Response of peer relations and social activities to treatment with viloxazine extended‐release capsules (Qelbree^®^): A post hoc analysis of four randomized clinical trials of children and adolescents with attention‐deficit/hyperactivity disorder

**DOI:** 10.1002/brb3.2910

**Published:** 2023-02-27

**Authors:** Stephen V. Faraone, Roberto Gomeni, Joseph T. Hull, Gregory D. Busse, Brendan Lujan, Jonathan Rubin, Azmi Nasser

**Affiliations:** ^1^ Departments of Psychiatry and of Neuroscience and Physiology SUNY Upstate Medical University Syracuse New York USA; ^2^ Pharmacometrica, Lieu‐dit Longcol La Fouillade France; ^3^ Supernus Pharmaceuticals, Inc. Rockville Maryland USA

**Keywords:** attention‐deficit/hyperactivity disorder (ADHD), peer relations, Qelbree^®^ (viloxazine extended‐release capsules), social activities

## Abstract

**Introduction:**

Attention‐deficit/hyperactivity disorder (ADHD) is associated with impairments related to peer relations (PR) and social activities (SA). The objective of this post hoc analysis was to assess the degree to which viloxazine extended‐release (viloxazine ER; viloxazine extended‐release capsules; Qelbree^®^) improves clinical assessments of PR and SA in children and adolescents with ADHD.

**Methods:**

Data were used from four Phase III placebo‐controlled trials of 100 to 600 mg/day of viloxazine ER (*N* = 1354; 6–17 years of age). PR and SA were measured with the Peer Relations content scale of the Conners 3rd Edition Parent Short Form's Peer Relation content scale (C3PS‐PR) and the Social Activities domain of the Weiss Functional Impairment Rating Scale‐Parent Report's (WFIRS‐P‐SA) at baseline and end of study. ADHD symptoms were assessed weekly with the ADHD Rating Scale, 5th Edition. The analyses relied on the general linear mixed model with the subject as a random effect.

**Results:**

Improvement in C3PS‐PR (*p* = .0035) and WFIRS‐P‐SA (*p* = .0029) scores were significantly greater in subjects treated with viloxazine ER compared with placebo. When using measures of clinically meaningful response, the C3PS‐PR responder rate was significantly higher for viloxazine ER (19.2%) compared with placebo (14.1%) and the difference was statistically significant (*p* = .0311); the Number Needed to Treat (NNT) was 19.6. The WFIRS‐P‐SA responder rate was significantly higher for viloxazine ER (43.2%) compared with placebo (28.5%) and the difference was statistically significant (*p* < .0001); the NNT was 6.8. The standardized mean difference effect size for both PR and SA was 0.09.

**Conclusions:**

Viloxazine ER significantly reduces the impairment of PR and SA in children and adolescents with ADHD. Although its effects on PR and SA are modest, many ADHD patients can be expected to achieve clinically meaningful improvements in PR and SA with viloxazine ER treatment for longer than 6 weeks.

## KEY POINTS

- Children and adolescents with attention‐deficit/hyperactivity disorder (ADHD) commonly experience deficits associated with peer relations (PR) and social activities (SA).
- Viloxazine extended‐release (viloxazine ER) is approved for the treatment of ADHD in children and adolescents.
- In a post hoc analysis of pooled data from four pediatric, Phase III, placebo‐controlled trials, viloxazine ER significantly improved the outcome in assessments of both PR and SA in children and adolescents with ADHD.

## 1 INTRODUCTION

Attention‐deficit/hyperactivity disorder (ADHD) is a common neurodevelopmental disorder that is often diagnosed in children (Faraone, Banaschewski, et al., 2021; Wolraich et al., 2019). Diagnosis of ADHD in children is based on clinician interviews with parents, teachers, and the patient regarding the presence of developmentally inappropriate levels of hyperactive‐impulsive and/or inattentive symptoms (Faraone, Banaschewski, et al., 2021; Wolraich et al., 2019). Presentation of ADHD can be primarily inattentive, primarily hyperactive‐impulsive, or combined (Faraone, Banaschewski, et al., 2021; Wolraich et al., 2019). ADHD is treated with both pharmacologic and nonpharmacologic methods (Cortese, 2020). The Food and Drug Administration (FDA) approved stimulant and nonstimulant pharmacologic therapies for treatment of ADHD in children (≥6 years) and adults (Cortese, 2020). These pharmacologic therapies have been shown to improve impairments associated with ADHD (Cortese, 2020; Faraone et al., 2015).

Impairments associated with ADHD include those of peer relations (PR) and social activities (SA). Children and adolescents with ADHD are at high risk for PR and SA impairments (Faraone et al., 2015; Faraone, Banaschewski, et al., 2021). In a study comprising over 8600 youths from the US National Health Interview Survey, ADHD children and adolescents (aged 4−17 years) were six times as likely to have a high level of peer problems, and nine times as likely to exhibit a high level of impairment concerning interference with home life, friendships, and leisure activities (Strine et al., 2006). In a meta‐analysis, which included data from 109 studies and over 104,000 children, ADHD was associated with an increased risk for clinically relevant impairments in socializing with peers, as measured by rejection/likability, popularity, and friendships (Ros & Graziano, 2018). ADHD children also had moderate impairments in social skills related to sharing, cooperating, turn‐taking, and reciprocity, as well as social information processing related to recognizing social cues, identifying problems, generating solutions, and avoiding biases (Ros & Graziano, 2018). Interestingly, meta‐analyses indicate that hyperactive‐impulsive symptoms, but not inattentive symptoms, are associated with peer rejection (Willcutt et al., 2012).

Some studies have assessed the impact of the medications used to treat the symptoms of ADHD on PR and SA in youth. Many of these studies have focused on stimulants, such as methylphenidate (MPH), which have generally been recommended as the first‐line therapy for ADHD (Cortese, 2020). In a double‐blind, placebo‐controlled study to investigate the effect of a single dose of MPH on social cognition, immediate‐release (IR) MPH treatment in ADHD children improved social cognition to a degree that was indistinguishable from untreated, typically developing, healthy controls (Levi‐Shachar et al., 2020). However, in a study of the long‐term effects of stimulant treatment for ADHD, no effect of stimulants was found on prosocial behavior after 6 years of treatment (Schweren et al., 2019). In a 12‐week, open‐label study of children and adolescents with ADHD that investigated the effects of MPH on the symptoms of social phobia, MPH significantly improved the symptoms of social phobia, especially those that had been evident at school (Golubchik et al., 2014). Notably, the improvements in social phobia symptoms were positively correlated with the improvement in ADHD symptoms (Golubchik et al., 2014). A two‐yearlong study of children (aged 7−9 years) with ADHD that compared MPH treatment alone versus MPH combined with multimodal psychosocial treatment found that both groups improved on the parent, child, and teacher ratings of social functioning, and on rater observations of behavior at school (Abikoff et al., 2004). However, no added benefit was reported in social functioning in the group receiving psychosocial treatment in combination with MPH relative to MPH alone (Abikoff et al., 2004). Pelham et al. (1987) compared the effects of sustained‐release MPH and IR MPH on social behavior in children with ADHD attending a summer camp program, wherein both drugs improved social behaviors and exhibited similar time courses of onset (Pelham et al., 1987). A 24‐week, open‐label clinical trial reported that osmotic‐release oral system MPH led to improvements in PR (parent report: Cohen's *d* = −.50; self‐report: Cohen's *d* = −.25) (Shang et al., 2020).

Other studies have focused on nonstimulant medications, including atomoxetine (ATX), as they have also been shown to be efficacious in treating ADHD (Cortese, 2020). The studies that have assessed the impact of nonstimulants on PR and SA have produced mixed results. Shang et al.’s 24‐week, open‐label trial reported that treatment with ATX led to improvements in PR (parent report: Cohen's *d* = −.33; self‐report: Cohen's *d* = −.65). Moreover, a placebo‐controlled trial of ATX with 297 children and adolescents reported that ATX improved psychosocial scores, as measured by the parent‐rated Child Health Questionnaire (Michelson et al., 2001). In a 14‐week randomized trial of ATX in adults with ADHD and social anxiety disorder, ATX significantly improved both the symptoms of ADHD and the symptoms of social anxiety disorder (Adler et al., 2009). However, an 8‐week, double‐blind, placebo‐controlled study of ATX in 97 children and adolescent patients with comorbid ADHD and autism spectrum disorders reported no beneficial effects of ATX on social functioning (Harfterkamp et al., 2014). Likewise, two other placebo‐controlled trials of ATX in adults reported that ATX did not improve ratings of Social Life on the Sheehan Disability Scale (Michelson et al., 2003).

Viloxazine extended‐release (viloxazine ER; viloxazine extended‐release capsules; Qelbree^®^) is a novel nonstimulant recently approved for the treatment of ADHD in adults and children ≥6 years of age (Nasser et al., 2022; Nasser et al., 2020; Nasser, Liranso, Adewole, Fry, et al., 2021a, 2021b; Nasser, Liranso, Adewole, Fry, Hull, Busse, et al., 2021). Viloxazine ER is a norepinephrine reuptake inhibitor; however, contemporary preclinical studies indicate that it also acts on serotonergic signaling in the brain, but the translational effect in humans remains to be fully elucidated (Yu et al., 2020). A series of Phase III clinical trials have been completed for viloxazine ER demonstrating its safety and efficacy in reducing the symptoms of ADHD in children and adolescents (Nasser et al., 2020; Nasser, Liranso, Adewole, Fry, et al., 2021a; Liranso, Adewole, Fry, Hull, Busse, et al., 2021). Moreover, a recent study in pediatric ADHD subjects demonstrated that the effect of viloxazine ER treatment on ADHD‐related functional impairment provides clinically meaningful improvements (Faraone, Gomeni, Hull, Busse, Melyan, Rubin, et al., 2021; Nasser, Liranso, Busse, et al., 2021).

From prior studies of other ADHD medications, there is no definitive evidence indicating whether these medications can improve PR and SA, although there does seem to be a consistent effect regarding improvements in symptoms of social phobia. The objective of the current study was to determine whether and to what degree viloxazine ER reduces the impairment of PR and SA in children and adolescents with ADHD. PR and SA were evaluated using the Peer Relations content scale of the Conners 3rd Edition Parent Short Form's Peer Relation (C3PS‐PR) *T*‐score and the Social Activities domain average score of the Weiss Functional Impairment Rating Scale‐Parent Report's (WFIRS‐P‐SA) respectively. This is the first study to assess if viloxazine ER improves PR and SA in children and adolescents with ADHD. Furthermore, this study had a large enough sample size to assess the magnitude of response in subjects showing severe levels of PR and SA at baseline. Clinically useful descriptions of response rates were provided by using a normed scale (the Conners 3), estimating the Number Needed to Treat (NNT), and assessing the subject's response in either PR or SA. In this manner, we sought to provide a more comprehensive evaluation of the PR and SA response than prior studies.

## 2 METHODS

The methods outlined here have been described previously (Faraone, Gomeni, Hull, Busse, Melyan, Rubin, et al., 2021).

### 2.1 Data description

Data was used from four Phase III, randomized, placebo‐controlled, double‐blind, 3‐arm, parallel‐group clinical trials of viloxazine ER in children and adolescents (aged 6−17 years) with ADHD (**Table** **1**) (Nasser et al., 2020; Nasser, Liranso, Adewole, Fry, et al., 2021a, 2021b; Nasser, Liranso, Adewole, Fry, Hull, Busse, et al., 2021). The study protocols were approved by Advarra Institutional Review Board (IRB) and conducted in accordance with the Helsinki Declaration and the International Council for Harmonisation Note for Guidance on Good Clinical Practice. All versions of the informed consent form were reviewed and approved by the IRB.

**TABLE 1:** Overview of Phase III randomized controlled trials providing data

Age group	Children (6−11 years of age)		Adolescents (12−17 years of age)	
Study ID No.	812P301	812P303	812P302	812P304
ClinicalTrials.gov identifier	NCT03247530	NCT03247543	NCT03247517	NCT03247556
No. of study sites^a^	34	28	33	27
Viloxazine ER doses (mg)^b^	100, 200	200, 400	200, 400	400, 600
Weeks, T + M	6 (1+5)	8 (3+5)	6 (1+5)	7 (2+5)
*N* randomized	477	313	310	297
*N* safety population	474	310	308	296
*N* ITT population	460	301	301	292
Viloxazine ER/placebo^c^	305/155	204/97	197/104	196/96

^a^ Study sites located in the United States.

^b^ Once‐daily dosing.

^c^ *N* based on ITT population.

Abbreviations: ITT, intent‐to‐treat; M, maintenance; *N*, number of subjects; *T*, titration; viloxazine ER, viloxazine extended‐release capsules.

### 2.2 Inclusion/exclusion criteria

In each study, screening began with signing the informed consent (and subject assent, if applicable). Eligibility was determined based on the predetermined inclusion/exclusion criteria. Subjects with a diagnosis of ADHD based on the Diagnostic and Statistical Manual of Mental Disorders, Fifth Edition criteria and confirmed by the Mini International Neuropsychiatric Interview for Children and Adolescents, ADHD Rating Scale, 5^th^ Edition (ADHD‐RS‐5) total score ≥ 28, and Clinical Global Impression‐Severity of Illness score ≥ 4 were eligible to participate.

Key exclusion criteria were: major psychiatric disorder or neurological disorder (excluding oppositional defiant disorder, or major depressive disorder if the subject was free of major depressive episodes both currently and for the 6 months prior to screening), a history of allergic reaction to viloxazine or its excipients, any food allergy or intolerance that contraindicated trial participation, suicidal ideation, history of seizures, significant systemic disease, or body mass index (BMI) > 95th percentile. Children and adolescents had to weigh ≤ 20 kg and ≤ 35 kg, respectively, and have BMI ≤ 95th percentile for the appropriate age and gender.

### 2.3 Randomization and treatment

After a screening period of up to 28 days, including a 7‐day washout period of prohibited medications, eligible subjects were randomized in a 1:1:1 ratio to receive one of the two doses of viloxazine ER or placebo. Subjects were instructed to take the study medication capsules daily by mouth in the morning, with or without food, throughout the treatment period. The viloxazine ER and placebo capsules were identical in appearance. If necessary, the subject's parent(s) or legal guardian(s) could open the viloxazine extended‐release capsules and sprinkle the contents over a spoon of soft food (e.g., applesauce) for consumption. Subjects were required to refrain from taking ADHD medications (other than the study medication) starting at least 1 week before randomization and throughout the study until the end of study (EOS).

### 2.4 Evaluations

Subjects returned weekly for efficacy and safety assessments until EOS or early termination (ET). ADHD‐RS‐5 was measured at the screening visit, baseline visit, and weekly postbaseline visits until EOS/ET. The C3PS and WFIRS‐P were administered at baseline and the EOS. The C3PS is a diagnostic tool for the assessment of ADHD and associated learning, behavioral, and emotional problems that have been validated in children and adolescents (Sparrow, 2010). The C3PS is completed by a child's parent/guardian and comprises 45 items with subsets of items related to six content scales: inattention, hyperactivity/impulsivity, executive functioning, learning problems, defiance/aggression, and PR. The C3PS generates a *T*‐score for each of the six content scales by scoring 43 of 45 items on a 4‐point Likert scale over the past month. The WFIRS‐P is an instrument that evaluates ADHD‐related functional impairment and has been psychometrically validated in children and adolescents aged 6 to 18 years (Gajria et al., 2015; Thompson et al., 2017). The WFIRS‐P is completed by the child's parent/guardian using 50 items that are grouped into six domains: family, school learning and behavior, life skills, child's self‐concept, SA, and risky activities. The 50 items are scored on a 4‐point Likert scale over the past month with the total score being the average for all 50 items and the domain score being the mean score of the items in that domain. PR and SA were assessed with the C3PS‐PR content scale *T*‐score and the WFIRS‐P‐SA domain average score, respectively.

### 2.5 Data analyses

The analyses used the general linear mixed model with the subject as a random effect and other independent variables as fixed effects using proc mixed procedure (SAS version 9.4). The first analysis included all subjects with C3PS‐PR/WFIRS‐P‐SA at EOS as the dependent variable. The following fixed effects were included as independent variables: C3PS‐PR/WFIRS‐P‐SA at baseline, treatment group (viloxazine ER versus placebo), age, and sex. The *F*‐test was used to determine whether the means between the two groups were significantly different. Because some subjects did not exhibit PR or SA at baseline, the mixed model analysis was run again for the subset of subjects having a C3PS‐PR *T*‐score greater than 70 at baseline and a WFIRS‐P‐SA average score greater than the median (1.4) WFIRS‐P‐SA average score at baseline.

The response rates were determined for C3PS‐PR *T*‐score and WFIRS‐P‐SA average score, which were analyzed using the chi‐square test. The response rate was based on the percentage of subjects who were defined as responders. Subjects were defined as a C3PS‐PR responder if their C3PS‐PR *T*‐score was greater than 70 at baseline and less than 65 at the EOS. A *T*‐score is a standardized score normalized for age and gender. A *T*‐score of 70 was chosen as defining impairment, as it is 2 standard deviations from the population mean (*T*‐score of 50), which is a standard method of defining severe impairment using *T*‐scores (Sparrow, 2010). A *T*‐score of 65 is 1.5 standard deviations above the mean; improvement from a *T*‐score of > 70 to < 65 represents a change from a score in the Very Elevated range to the High Average range (Sparrow, 2010). Subjects were defined as a WFIRS‐P‐SA responder if their change from baseline (CFB) WFIRS‐P‐SA average score was reduced by 50% or more based on our previous analysis, which established a WFIRS‐P‐SA score reduction of 43% in children and adolescents as a clinically meaningful improvement (Nasser, Kosheleff, et al., 2021). The ADHD‐RS‐5 data from the four pivotal Phase III clinical trials were integrated with a cutoff of 6 weeks of treatment as the common efficacy endpoint (Nasser, Liranso, Adewole, Fry, Hull, Busse, et al., 2021; Nasser et al., 2020; Nasser, Liranso, Adewole, Fry, et al., 2021a, 2021b).

## 3 RESULTS

The mean C3PS‐PR *T*‐scores and WFIRS‐P‐SA average scores at baseline and EOS for both the viloxazine ER and placebo study groups are displayed in **Table** **2** (Data sets 1−4, 2018−2019).

**TABLE 2:** Mean (± SD) C3PS‐PR *T*‐score and WFIRS‐P‐SA average score at baseline and EOS

Measure	Visit	Placebo (*N* = 394)	Viloxazine ER (*N* = 760)
C3PS‐PR (mean ± SD)	Baseline	70.9 ± 17.6	70.8 ± 17.5
	EOS	66.2 ± 18.1	63.6 ± 17.2
WFIRS‐P‐SA (mean ± SD)	Baseline	0.9 ± 0.8	0.9 ± 0.7
	EOS	0.6 ± 0.7	0.5 ± 0.6

Abbreviations: C3PS‐PR, Conners 3rd Edition Parent Short Form; EOS, end of study; *N*, number of subjects; SD, standard deviation; viloxazine ER, viloxazine extended‐release capsules; WFIRS‐P‐SA, Weiss Functional Impairments Scale‐Parent Report's Social Activities domain.

The mean baseline C3PS‐PR *T*‐score was in the severe range for both viloxazine ER and placebo study groups (Sparrow, 2010). With C3PS‐PR score as the outcome, there was a significant effect for baseline C3PS‐PR score (*F*_1, 1150_ = 323, where 1 and 1150 represent the degrees of freedom of the *F* value numerator [variance between groups] and denominator [variance within groups], respectively; *p* < .0001) and viloxazine ER treatment group (*F*_1,1150_ = 8.5, *p* = .0035) (Data sets 1−4, 2018−2019). Age and sex were not significant. The CFB in the C3PS‐PR *T*‐score (least square [LS] means ± standard error [SE]) was greater in the viloxazine ER group (7.26 ± 0.52) compared to placebo (4.65 ± 0.73), and the difference (2.61 ± 0.89) was statistically significant (*p* = .0035; **Table** **3**) (Data sets 1−4, 2018−2019).

**TABLE 3:** CFB in the peer relations and social activities scores (LS means estimated in the general linear mixed effects models)

	CFB in C3PS‐PR *T*‐score, LS means					
	All subjects			Subjects with baseline *T*‐score > 70		
Treatment	CFB	SE	*p* Value	CFB	SE	*p* Value
**Placebo**	4.65	0.73		10.51	1.10	
**Viloxazine ER**	7.26	0.52		14.21	0.79	
**Viloxazine ER − placebo**	2.61	0.89	.0035	3.70	1.35	.0063

	CFB in the WFIRS‐P‐SA average score, LS means					
	All subjects			Subjects with baseline average score > 1.4		
**Treatment**	CFB	SE	*p* Value	CFB	SE	*p* Value
**Placebo**	0.23	0.03		0.68	0.02	
**Viloxazine ER**	0.33	0.02		0.74	0.01	
**Viloxazine ER − placebo**	0.10	0.03	.0029	0.06	0.03	.0155

Abbreviations: C3PS‐PR, Conners 3rd Edition Parent Short Form's Peer Relations content scale; CFB, change from baseline; LS, least squares; SE, standard error; viloxazine ER, viloxazine extended‐release capsules; WFIRS‐P‐SA, Weiss Functional Impairments Scale Parent Report's Social Activities domain.

When limiting the analysis to those subjects with a C3PS‐PR baseline score greater than 70, a significant effect was found for baseline C3PS‐PR score (*F*_1,621_ = 7.4, *p* = .0068) and viloxazine ER treatment group (*F*_1,621_ = 7.5, *p* = .0063) (Data sets 1−4, 2018−2019). Age and sex were not significant. The CFB in the C3PS‐PR *T*‐score (LS means ± SE) was greater in the viloxazine ER group (14.21 ± 0.79) compared to placebo (10.51 ± 1.10), and the difference (3.70 ± 1.35) was statistically significant (*p* = .0063; **Table** **3**) (Data sets 1−4, 2018−2019).

With WFIRS‐P‐SA score as the outcome, there was a significant effect for baseline WFIRS‐P‐SA score (*F*_1, 1141_ = 483, *p* < .0001) and viloxazine ER treatment group (*F*_1, 1141_ = 8.9, *p* = .0029) (Data sets 1−4, 2018−2019). Age and sex were not significant. The CFB in the WFIRS‐P‐SA average score (LS means ± SE) was greater in the viloxazine ER group (0.33 ± 0.02) compared to placebo (0.23 ± 0.03), and the difference (0.10 ± 0.03) was statistically significant (*p* = .0029; **Table** **3**) (Data sets 1−4, 2018−2019).

When limiting the analysis to those subjects with a WFIRS‐P‐SA baseline score greater than the median (1.4), a significant effect was found for baseline WFIRS‐P‐SA score (*F*_1,566_ = 1335, *p* < .0001) and viloxazine ER treatment group (*F*_1, 566_ = 5.9, *p* = .0155) (Data sets 1−4, 2018−2019). Age and sex were not significant. The CFB in the WFIRS‐P‐SA average score (LS means ± SE) was greater in the viloxazine ER group (0.74 ± 0.01) compared to placebo (0.68 ± 0.02), and the difference (0.06 ± 0.03) was statistically significant (*p* = .0155; **Table** **3**) (Data sets 1−4, 2018−2019).

**FIGURE 1:**
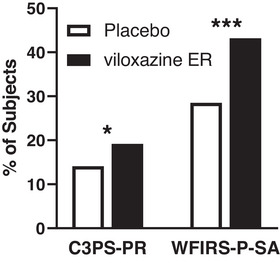
The impact of viloxazine ER on peer relations and social activities by responder analysis. Responder rate between viloxazine ER and placebo groups for C3PS‐PR and WFIRS‐P‐SA are displayed. A C3PS‐PR responder was defined as a subject who had a C3PS‐PR *T*‐score greater than 70 at baseline and less than 65 at the EOS. A WFIRS‐P‐SA responder was defined as a subject who had a 50% or greater reduction in the WFIRS‐P‐SA average score from baseline to the EOS. (*p < .05, ***p < .001). C3PS‐PR, Conners 3rd Edition Parent Short Form's Peer Relation; EOS, end of study; viloxazine ER, viloxazine extended‐release capsules; WFIRS‐P‐SA, Weiss Functional Impairments Scale Parent Report's Social Activities domain.

The effect of viloxazine ER on C3PS‐PR *T*‐score and WFIRS‐P‐SA average score was analyzed by responder analysis and is presented in **Figure** **1** (Data sets 1−4, 2018−2019). When defining response as a subject who had a C3PS‐PR *T*‐score greater than 70 at baseline and less than 65 at EOS, the C3PS‐PR responder rate was higher in viloxazine ER (19.2%) compared with placebo (14.1%), and the difference was statistically significant (χ^2^_1_ = 4.6, *p* = .0311); the NNT was 19.6 (Data sets 1−4, 2018−2019). When defining response as a 50% or greater decrease in the WFIRS‐P‐SA average score from baseline to EOS, the responder rate was higher in viloxazine ER (43.2%) compared with placebo (28.5%), and the difference was statistically significant (χ^2^_1_ = 23.6, *p* < .0001); the NNT was 6.8 (Data sets 1−4, 2018−2019).

**FIGURE 2:**
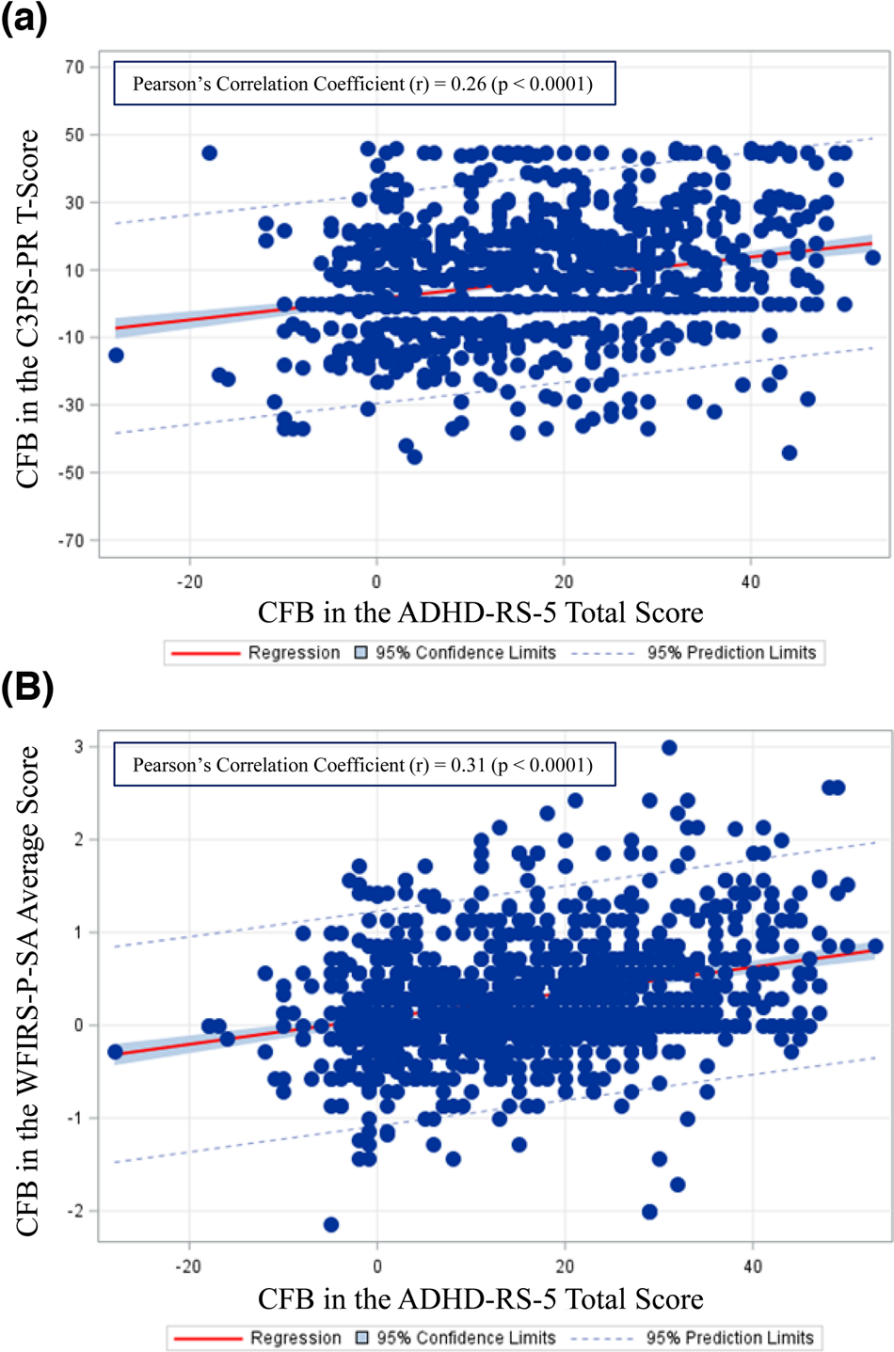
Association between the CFB in peer relations or social activities scores and the CFB in the ADHD‐RS‐5 total score with viloxazine ER treatment. Positive scores denote improvement. The CFB in ADHD‐RS‐5 total score is plotted as a function of the CFB in C3PS‐PR *T*‐score (A) or WFIRS‐P‐SA average score (B). Linear regression analysis was performed, and the associated 95% confidence limits and 95% prediction limits are displayed. For C3PS‐PR and WFIRS‐P‐SA, CFB = Baseline score − score at EOS; for ADHD‐RS‐5, CFB = Baseline score − score at Week 6. ADHD‐RS‐5, ADHD Rating Scale, 5th Edition; C3PS‐PR, Conners 3rd Edition Parent Short Form's Peer Relation; CFB, change from baseline; EOS, end of study; viloxazine ER, viloxazine extended‐release capsules; WFIRS‐P‐SA, Weiss Functional Impairments Scale Parent Report's Social Activities domain.

Among viloxazine ER‐treated subjects, the correlation between the magnitude of the C3PS‐PR *T*‐score response and the magnitude of ADHD‐RS‐5 symptom response produced a Pearson's correlation coefficient (*r*) of 0.26 (*p* < .0001) (Data sets 1−4, 2018−2019). The corresponding correlation for the WFIRS‐P‐SA average score was 0.31 (*p* < .0001) (Data sets 1−4, 2018−2019). **Figure** **2** shows the scatterplots corresponding to these two correlation coefficients.

## 4 DISCUSSION

In a series of pivotal Phase III trials in children and adolescents, viloxazine ER was demonstrated to reduce ADHD symptoms, evaluated with the ADHD‐RS‐5, in as little as 1 week of treatment (Nasser et al., 2020; Nasser, Liranso, Adewole, Fry, et al., 2021a; Nasser, Liranso, Adewole, Fry, Hull, Busse, et al., 2021). Using the C3PS‐PR content scale *T*‐score and WFIRS‐P‐SA domain average score, the results of this post hoc analysis indicated that viloxazine ER treatment produced statistically significant reductions in the impairment associated with PR (*p* = .0035) and SA (*p* = .0029) versus placebo in pediatric subjects with ADHD (Data sets 1−4, 2018−2019). Contrary to our expectations, the effect of viloxazine ER was not much greater when we limited our analysis to those subjects that exhibited severe levels of PR and SA at baseline, possibly due to our sample having high PR and SA scores at baseline (**Table** **2**).

The effect of viloxazine ER on the WFIRS‐P‐SA average score was more robust than the effect of viloxazine ER on the C3PS‐PR *T*‐score (Data sets 1−4, 2018−2019). One potential explanation is the item content of each subscale. For instance, the C3PS‐PR items focus on the child's social engagement with peers. Example items are “Has trouble keeping friends” and “Has no friends.” The WFIRS‐P‐SA has similar items that overlap with the C3PS‐PR, but additionally contains items that focus on problem behaviors such as “Teases or bullies other children” and “Problems getting along with other children.” Other clinical trials have shown that the medications used to treat the symptoms of ADHD are modestly efficacious for such disruptive and aggressive behaviors (Pringsheim et al., 2015). We should also keep in mind that a short‐term treatment trial may not be sufficient to capture all PR and SA improvements. For example, the C3PS‐PR item “Has no friends” is unlikely to change over a trial duration of 6−8 weeks. Thus, exploration of the impact of viloxazine ER on PR and SA in a longer‐term study could be examined in the future.

**Figure** **2** shows that, although there is a significant correlation between the change in C3PS‐PR *T*‐score or the WFIRS‐P‐SA average score and the change in ADHD‐RS‐5 symptom score, some subjects improve more in one area than the other (Data sets 1−4, 2018−2019). For example, many subjects who improve more than 20 points (2 standard deviations) on the C3PS‐PR *T*‐score do not show an improvement in the ADHD‐RS‐5 symptom score, and some subjects with a 40‐point improvement in ADHD‐RS‐5 symptom score show little or no improvement in the C3PS‐PR *T*‐score (Data sets 1−4, 2018−2019). **Figure** **2** also demonstrates that many subjects show large improvements in both the C3PS‐PR *T*‐score and ADHD‐RS‐5 symptom score (Data sets 1−4, 2018−2019). For example, the scatterplot shows that many subjects improve more than 20 points (2 standard deviations) on the C3PS‐PR *T*‐score and also show an improvement of 20 or more points on the ADHD‐RS‐5 symptom score (Data sets 1−4, 2018−2019). We saw similar results for the WFIRS‐P‐SA average score (Data sets 1−4, 2018−2019). These observations of differential response are consistent with our finding that, although the correlation between ADHD symptom change and PR/SA change with treatment is statistically significant, the correlations are low. The change in ADHD symptoms shares only 7% of variance with change in C3PS‐PR scores and only 10% of variance with change in WFIRS‐P‐SA scores (Data sets 1−4, 2018−2019).

Given these results, how should clinicians think about the use of viloxazine ER for the treatment of PR and SA in ADHD pediatric populations? For viloxazine ER, about 43% of patients should show clinically significant improvement (≥ 50% reduction CFB) in SA, as measured by the WFIRS‐P‐SA. This response rate corresponds to an NNT of 6.8, which shows that, in addition to the benefit viloxazine ER affords for reducing the symptoms of ADHD (Nasser et al., 2020; Nasser, Liranso, Adewole, Fry, et al., 2021a; Nasser, Liranso, Adewole, Fry, Hull, Busse, et al., 2021), some patients will experience substantial improvements in social functioning. Prior evidence indicates mixed effects of the medications used to treat the symptoms of ADHD on PR and SA. While some studies of MPH treatment in children with ADHD demonstrated improvement in social cognition, social phobia, and social functioning (Abikoff et al., 2004; Golubchik et al., 2014; Levi‐Shachar et al., 2020), a 6‐year study found no effect of stimulant treatment for ADHD on prosocial behavior (Schweren et al., 2019). Similarly, some studies of ATX treatment for ADHD in children, adolescents, or adults found improvement in PR, psychosocial scores, and social anxiety disorder symptoms (Adler et al., 2009; Michelson et al., 2001; Shang et al., 2020), while others found no effect on social functioning (Harfterkamp et al., 2014; Michelson et al., 2003). In the current analysis of relatively short‐term trials, the effect size for both PR and SA was 0.09 (Data sets 1−4, 2018−2019). Though this is a relatively small effect size, these data are from short‐term trials that were powered to assess the primary endpoint, not SA and PR. This small effect size is unlikely to be attributable to a placebo effect, as there were statistically significant differences in responder rates for both SA and PR measures also reflected by the NNTs. Further long‐term studies are needed to confirm the effects seen and rule out any placebo effects. Although we do not have long‐term data on the effects of viloxazine ER on social functioning, one would expect those improvements in social functioning to lag behind observed improvements in ADHD symptoms. The attractiveness of viloxazine ER as a nonstimulant option is further increased by a prior report showing that ADHD symptom response at Week 6 can be predicted, with reasonable accuracy, after 2 weeks of treatment (Faraone, Gomeni, Hull, Busse, Melyan, O'Neal, et al., 2021).

The mechanism by which ADHD medications, including viloxazine ER, may improve PR and SA is unknown. However, the correlation analysis (**Figure** 2) shows that viloxazine ER improvement of overall ADHD symptoms is correlated with improvement in PR and SA. Results of our previous pediatric studies support this idea, as viloxazine ER treatment in children and adolescents improved overall ADHD symptoms in conjunction with family and peer relationships (Nasser, Liranso, Busse, et al., 2021). In addition, a study in adults with ADHD found treatment with MPH and ATX improved ADHD symptoms as well as relationships and social impairments (Ni et al., 2017). Thus, improvement in overall ADHD symptomology may allow for improvement in domains impacted by these symptoms, including PR and SA.

The work presented here should be evaluated in the context of some limitations. Because behavioral measures of peer relations and social activities were used, these results collected as part of clinical trials may not generalize to “real‐world” measures, sociometric ratings, and in vivo behavioral observations. A potentially confounding factor is that the subjects’ parents completed the questionnaires used here due to the age of the subjects. Although this could have induced spurious correlations among rating scales, use of parent ratings could not have induced drug versus placebo differences, given that subjects were randomized to these groups. These findings may not generalize to other parent‐reported outcome measures of PR and SA. Indeed, the differences in results for PR on the one hand and SA on the other (in the present study and the literature) suggest that these two constructs be evaluated separately. Despite these limitations, we can conclude that viloxazine ER significantly reduces PR and SA in pediatric subjects with ADHD. Although the average effects of viloxazine ER on PR and SA are modest, a substantial minority of patients will achieve clinically significant improvements in PR and SA.

## CONFLICT OF INTEREST STATEMENT

JTH, GDB, and JR are employees of Supernus Pharmaceuticals, Inc. BL and AN were employees of Supernus Pharmaceuticals, Inc. at the time the study was conducted. SVF received income, potential income, travel expenses, continuing education support, and/or research support from Aardvark, Rhodes, OnDosis, Tris, Otsuka, Arbor, Ironshore, KemPharm/Corium, Akili, Supernus, Takeda, Atentiv, and Genomind. With his institution, he has US patent US20130217707 A1 for the use of sodium‐hydrogen exchange inhibitors in the treatment of ADHD. He also receives royalties from books published by Guilford Press: *Straight Talk about Your Child's Mental Health*; Oxford University Press: *Schizophrenia: The Facts*; and Elsevier: *ADHD: Non‐Pharmacologic Interventions*. He is Program Director of www.adhdinadults.com. RG was a paid consultant to Ironshore Pharmaceuticals, Sunovion Pharmaceuticals, Supernus Pharmaceuticals, Teva, Biomedical Science Institutes, Nanomi BVs, Laboratorios Liconsa, Massachusetts General Hospital, UCB, Recordati Rare Diseases, Indivior, Tris Pharma, and F. Hoffmann‐La Roche.

### PEER REVIEW

The peer review history for this article is available at https://publons.com/publon/10.1002/brb3.2910.

## ETHICS STATEMENT

The study protocols were approved by Advarra IRB and conducted in accordance with the Helsinki Declaration and the International Council for Harmonisation Note for Guidance on Good Clinical Practice. All versions of the informed consent form were reviewed and approved by the IRB.

## CLINICAL TRIAL REGISTRATION

NCT03247530, NCT03247517, NCT03247543, NCT03247556.

## Data Availability

These data are not available in a repository, but reasonable requests can be sent to Azmi Nasser at anasser@supernus.com.
